# The use of public spatial databases in risk analysis: A US‐oriented tutorial

**DOI:** 10.1111/risa.17705

**Published:** 2025-01-18

**Authors:** Michael R. Greenberg, Dona Schneider, Louis Anthony Cox

**Affiliations:** ^1^ Edward J. Bloustein School of Planning and Public Policy Rutgers The State University of New Jersey New Brunswick New Jersey USA; ^2^ Cox Associates, LLC Denver Colorado USA

**Keywords:** accuracy, databases, environmental justice, risk, scale/shape

## Abstract

This tutorial focuses on opportunities and challenges associated with using six large, publicly accessible spatial databases published during the last decade by US federal agencies. These databases provide opportunities for researchers to risk‐inform policy by comparing community asset, demographic, economic, and social data, along with anthropogenic and natural hazard data at multiple geographic scales. The opportunities for data analysis come with challenges, including data accuracy, variations in the shape and size of data cells, spatial autocorrelation, and other issues endemic to spatial datasets. If ignored, these issues can lead to misleading results. This article briefly reviews the six databases and how agencies use them. It then focuses on the data and its limitations. Examples are provided, as are summaries of the debates surrounding these databases, followed by paths forward for improving their use. We end with a checklist that users should consider when they access any of the six spatial databases or others. We believe that these new resources can be effectively used with appropriate caution to answer user‐generated questions about hazards and risks—questions that are important to both community groups and government decision‐makers.

## 1 INTRODUCTION

In the last decade, US federal government agencies and departments, as well as several states and non‐governmental organizations, published spatial databases accessible to anyone who has a laptop, tablet, or even a hand‐held device. The databases cover hundreds of variables, allowing users to see and analyze the geographical distribution of many indicators of hazards and risk at granular levels. Although database managers have tried to alert potential users about the limitations of their databases, they have not always been successful. For example, the US Environmental Protection Agency's (U.S. EPA, n.d.‐a) EJScreen documents every variable in its database, and the University of Wisconsin, Population Health Institute (n.d.) does the same, explaining how each fits into its overall model of health outcomes (see Section 3 for more detail). However, we have observed that after students are exposed to these resources, they become empowered and often excited by learning about their neighborhoods and other areas of interest. They enjoy generating maps accompanied by tables and other displays. Unfortunately, some jump to conclusions without reading, absorbing, and understanding the database's cautionary notes.

This tutorial addresses the need for risk analysis practitioners, whether students or professionals, to simultaneously appreciate how public spatial databases can be used to address risk analysis questions while being aware of their limitations. Our intended scope and major emphasis is on using these databases to inform public policy, and our secondary focus is on their use for teaching. We first introduce six large US databases that provide information at multiple geographic scales, beginning by summarizing them and reviewing their data accuracy issues. Following is a review of how these databases are used by their host organizations, providing additional examples of their uses by analysts. A penultimate section identifies challenges and opportunities potential users will likely encounter and offers suggestions about accessing information about how to handle these issues. The final section provides tips for spatial database users.

Overall, we view this tutorial as a bridge between rapidly improving public access to data and the ongoing challenge of producing information that can be used to risk‐inform decisions rather than direct them. The pressure on the creators of these databases has been elevated by federal programs such as the American Inflation Reduction Act and Justice40, as well as by state, local, and private organizations that partly depend on the information to inform their decisions. Given the space limitations of journal articles, the text discusses some of these challenges while the tables highlight others.

## 2 THE SIX DATABASES

More than 30 relatively new databases offer information for US counties, municipalities, census tracts, and other small areas. The number of these databases continues to increase, especially for state governments (e.g., California and Maryland) and specialty risk‐related areas, such as transportation (Federal Highway Administration, n.d.; Federal Railway Administration, 2023; Lievanos, 2018; U.S. Department of Transportation, 2023; Williams et al., 2022). This tutorial features six databases that offer information for every area of the United States. They include a variety of risk‐related variables, and at least two of the authors of this article have used them in their research. The six also cover a broad set of anthropogenic and natural hazards and risk management policies, and they are accompanied by explanations of technical information (Table 1). Although other interesting databases provide data for a specific place (e.g., Maryland, California) or issue (e.g., transportation), the six covered in this tutorial provide the background for discussing the applicability of spatial data for risk‐informing public policy across the country.

**TABLE 1:** Six large risk‐related U.S. databases.

Database	US department or agency	Year released	Data types	Geographic scale
County Health Rankings and Roadmaps	University of Wisconsin Population Health Institute	2010	Primarily health outcomes and behaviors, also demographic, social, economic, and environment	County, state, and national
EJScreen	US Environmental Protection Agency	2015	Primarily environmental, also demographic, social, economic, infrastructure, and other services	Census block/tract, municipality, county, state, national, and user‐created polygons
PLACES	Centers for Disease Control and Prevention & Robert Wood Johnson Foundation	2015	Health outcomes and factors influencing outcomes	Census tract, municipality, state, and national
National Risk Index	Federal Emergency Management Agency	2021	Focus on the economic impacts of 18 natural hazards	Census tract, county, state, and national
Environmental Justice Index	Center for Disease Control and Prevention, Office of Environmental Justice & ATSDR	2022	A mixture of environmental, health, social, economic, and demographic indicators intended to measure cumulative risk for environmental justice	Census tract, state, and county
Climate and Environmental Justice Screening Tool	Council on Environmental Quality	2022	Mixture of environment, health, social, economic, demographic indicators intended to measure cumulative risk for Justice40 eligibility	Census tract

County Health Rankings and Roadmaps (CHRR) was developed by the University of Wisconsin to support state, county, and municipal health planners in the United States. Data for counties are available from 2010. Hence, CHRR is an excellent database where users can explore spatial and temporal patterns. The creator offers seminars on a variety of health‐related topics and includes practical suggestions for improving their use (University of Wisconsin, Population Health Institute, n.d.). CHRR is a useful teaching tool, especially for current and future health professionals, as well as analysts who operate at the county scale.

EJScreen was created by the U.S. EPA (n.d.‐a) as a response to President Clinton's 1994 Environmental Justice and Executive Order 12898. That order directed federal agencies to identify and address disproportionately high and adverse environmental or human health effects on low‐income and minority populations. First released in 2015, EJScreen has steadily improved by adding new variables and ways of displaying information and by allowing users flexibility in examining the data. For example, users can create polygons from block group data. This is important when risk follows a narrow path, such as a highway. The geographic information system (GIS) tool automatically allocates data to places using a kriging (spatial linear regression) method or other calculations that the EPA explains (U.S. EPA, n.d.‐b). EJScreen is extremely popular with students who enjoy examining their neighborhoods and adjacent ones for similarities and differences. However, some of the data, especially the environmental data, should be cautiously interpreted for a specific place because the numbers are estimates based on limited monitoring data and mathematical models (see Section 3.3 for more detail).

The Center for Disease Control and Prevention's (CDC) PLACES database, like CHRR, was originally intended for local health officials. It provides data at the neighborhood scale. The first version concentrated on the 500 most populated cities in the United States and was accompanied by electronic books of maps comparing dozens of health outcomes and behavioral indicators (CDC, 2016). Current updates include new metrics and searchable maps (CDC, 2023). Thus, PLACES is a valuable set of tools for local health planning.

The Federal Emergency Management Agency's (FEMA) National Risk Index (NRI) (FEMA, 2024) is tied to the increasing cost of federal post‐natural disaster support of state and local governments. States and many local governments prepare hazard mitigation plans (normally updated every 5 years) to be eligible for federal disaster relief and planning money. The database uses census data and the broader hazards literature to inform state and local officials about the economic risks to their populations and constructed environments. It also estimates the resilience of areas should a natural hazard event occur, allowing comparisons with the host state and the nation. This provides important comparative information for planners and decision‐makers allocating resources to mitigate hazards.

CDC/ATSDR's Environmental Justice Index (EJI) was developed to create comparisons of relative cumulative risk for multiple health and environmental indicators at the census tract scale (see Section 4.5); that is, the user can compare cumulative risk in one area with others. Launched in 2022, the database uses information from the Census Bureau, EPA, and other federal agencies. It presents its conceptual and statistical approach for accumulating the information into a few risk indices. The current database is a good start, and we expect it to be refined as ATSDR hears from users and evaluates their experiences with it (CDC/ATSDR, n.d.).

The final database is the Climate and Economic Justice Screening Tool (CEJST), created by the US Council on Environmental Quality (CEQ, n.d.) in response to the Biden administration's Justice40 program. Justice40 calls for allocating 40% of selected federal government funds to needy communities (The White House, 2022). Federal agencies must use CEJST in their program allocation decisions, although they can also use their agency's data. Not surprisingly, when CEQ released its beta version for review, it received over 100 user suggestions. After making many of the suggested changes, CEQ reissued CEJST. Dean and Esling (2023) describe CEJST as “a small map, with big implications.” It is used by multiple federal agencies and incorporated into other databases, for example, EJScreen (see below).

## 3 ACCURACY AND SPATIAL STATISTICAL CHALLENGES

This section describes some challenges users may encounter when they use the six databases described above. It examines generic issues and then considers health and demographic information, along with environmental, natural hazards, and community asset data.

### 3.1 Generic issues

How accurate are the tabular and mapped data derived from the six databases? When the digital data era dawned, the US federal government recognized the potential for misinformation and, in 1982, created a National Committee for Digital Cartographic Data Standards (NCDCDS). That agency was charged with developing a list of data issues and setting protocols for managing them (Moellering, 1987). Below are seven generic questions about dataset accuracy and completeness as identified by NCDCDS and amended by us:

1. Source of data: Has the source of the information and its method of derivation (including any transformations and methods of dealing with rounding and missing data) been reported?
2. Positional accuracy: Have the results been compared to an independent source with higher accuracy?
3. Verification of data accuracy: Has the information been verified with independent sample data, or were the results derived by a deductive method?
4. Demonstration of logical consistency: Is there evidence that the results make sense (are coherent) as part of a chain of linked information?
5. Completeness: Are agreed‐upon definitions presented, and have protocols for mapping the information been followed?
6. Temporal accuracy: Are the time intervals for the data specified?
7. Shape and land use variation: Is the size and shape of the area suitable for analysis? Is much of the area undeveloped? Is it oddly shaped, or does it otherwise misrepresent the population, leading users to potentially misleading results? Is it possible to use the databases to build areas that overcome shape and size limitations?

Openshaw (1989) observes that we need to learn to live with a small number of random errors. But even small errors can cause serious problems for statistical inference if they tend to be in one direction, reflecting a systematic bias (Martinez‐Parrales et al., 2020). For example, over‐counting a local environmental hazard by accidentally attributing its location to more than one grid cell may be more likely than undercounting, in which the hazard is not assigned any location. Sampling locations concentrated in easily accessible areas can bias the data collected; inappropriate map projections or coordinate systems can distort estimates of the areas covered by fires, floods, or other large spatial events; and smoothing algorithms can systematically underestimate peak values of environmental parameters such as air pollution concentrations, affecting hazard assessments. When random errors in locations are found and reported, they are usually correctable. Deliberate mistakes are usually found and dealt with. Systematic errors may not be found, and these may bias results. When a systematic error is found and reported, the agency should warn users and regenerate the dataset.

The six databases collect data for areas, lines, and points and display it in tables and maps. They offer information at the county, municipal, and census tract scales and provide state and national data for comparisons. EJScreen allows users to build shapes that approximate roads and other shapes with buffers around them, which is an important attribute (see cannabis example below).

### 3.2 Health and demographic data

The most accurate data should be demographic, health, and other data collected for the population or a sample of people in counties, municipalities, and census tracts by the US Census Bureau. Samples of income, race/ethnicity, age, and other data collected by the decennial census or by the annual American Community Surveys are calculated for the population of every area. These results should be considered credible. To protect privacy, when the population in an area is less than a threshold, the Census Bureau will report a population count but not population characteristics, such as median income and education. Some respondents refuse to fill out or selectively fill out census forms. This will likely bias the results in some places.

Databases that provide 95% or 90% confidence limits are an added asset. Much of the health data (e.g., mortality and morbidity) is reported as rates for counties, cities, and census tracts with 95% confidence limits. However, there are other ways of expressing variation around central tendencies, such as quartiles and deciles. In the 500 Cities project (later replaced by the PLACES project), the CDC recognized that small numbers of people in many census tracts would bias estimates for health outcomes and behaviors. To address the problem, the CDC used a data clustering method devised by Jenks (1967). The method reported census tract rates within ranges or classes and placed the numerical data into “natural” groups by identifying breakpoints to minimize the variance within clusters while maximizing the variance between them. This allows users to see how each tract compares to neighboring tracts without relying on a single estimated rate, which might be highly uncertain.

Overall, the reliability of data on health and population size is reduced when the geographic scale shifts to smaller scales. Accuracy is most uncertain in census tracts and small towns. Tracts have average populations of about 4000 but, in some cases, may be 2000 or less. Users must be aware of the uncertainty introduced by small population sizes in these databases.

### 3.3 Environmental data

As noted earlier, the environmental data used in EJScreen should be seen as approximations of environmental quality. Nearly all the air quality data have been collected from fixed monitors. As there are a limited number of sites that monitor PM2.5, ozone, and other national air quality indicators, data accuracy distal to these monitors is a problem. Many are located miles from the area they are representing. Some are located near urban intersections, whereas others are located in parks, differences that introduce location bias into the data. Some monitors are separated from the areas they represent by large manufactured or natural features. GIS tools use kriging (essentially a form of spatial linear regression) to estimate the values among the monitors, but this does not necessarily provide the same results as a monitor located on site. Such results rest on technical assumptions about the data‐generating process (e.g., stationarity, isotropy, or assumptions about anisotropy, known variogram, normality, or specified alternatives). The EPA uses statistical models to estimate values of carcinogenic, diesel, and other air pollutants for almost every location. The literature recognizes the limitations of these models, and studies suggest that portable monitors will slowly replace fixed monitoring stations (Knibbs et al., 2011). Yet, low‐cost portable monitors come with concerns about accuracy and equipment malfunctions. For example, Khreis et al. (2022) tested low‐cost monitors in Dallas, Texas, and reported differences in results from high‐accuracy regulatory monitors, as well as for mechanical failures resulting in loss of data. Until the data‐gathering process is improved significantly, conclusions about air quality burdens based on published data need to be viewed as approximations. Presumably, the cost will decrease with use, and scientists will learn enough to determine which of these portable monitors are trustworthy for different environments.

The EPA uses the distance from highways, hazardous industrial locations, and waste sites to the centroids of census tracts as a surrogate for potential exposures of people in those tracts. Such measurements should not be confused with actual exposures. U.S. EPA's (n.d.‐c) map descriptions explain how these numbers were calculated and their use as proxies. Other types of exposure estimates are made, as well. For example, exposure to lead is based on housing age, with the metric being the proportion of houses built before 1980 (the United States banned the use of lead paint in housing in 1978). Although the values for environmental lead should not be taken as actual exposures, they can perhaps be used collectively as a rough proxy for indoor lead environmental quality until more accurate data become available.

Data limitations are even wider when they concern drinking water and indoor air quality. The only variable commonly reported for drinking water is the absence or presence of a drinking water quality violation, and those reports are only for local government areas. For air quality, we know that people, especially the youngest and oldest of the population, spend nearly all of their time indoors. Unfortunately, public databases provide no information on indoor air quality.

A final note is that this section on environmental data should not be taken as a critique of the EPA. The agency is well aware of these data accuracy issues and has reported on the limitations and caveats inherent in using EJScreen (U.S. EPA, n.d.‐d). The agency has accomplished a good deal with limited funding for its spatial databases and could address more of these issues if additional resources were provided. Until the agency can add more specific data to its public databases, users should be aware of the data limitations.

### 3.4 Natural hazards

FEMA's natural hazard database estimates potential economic damage from tornadoes, floods, and more than a dozen other natural hazards that threaten people and property (FEMA, n.d.). Users will find that large urban centers and places with more wealth have more to lose but often have greater resources to help them recover from natural hazard events compared to rural and poor areas. Overall, the FEMA database, like the EPA one, provides valuable information to gain an overview of relative risk. Users, however, need to recognize that the published numbers are estimates.

### 3.5 Community assets

The U.S. EPA [Link], [Link], CEQ [Link], [Link], and University of Wisconsin, Population Health Institute (n.d.) databases include asset information, such as broadband networks, affordable housing, churches, hospitals, food venues, and other local service indicators. Some of these data are reported in tables and shown on maps. In each case, the locations are imprecise, as are the numbers of workers and service capacity. Some data can be retrieved directly from an agency through direct contact with staff at the federal, state, and city levels. Staff are busy, but our experience is that they will help users. Some available data includes the exact location of public housing assets, number of rooms, and estimated capacity. Nearest neighbor analysis tools (Getis, 1964) were developed to assess the clustering of these point datasets. However, we would not advise using the datasets for community assets unless you can secure precise data on their map coordinates.

Overall, how accurate is the information in these six databases? Although no definitive answer is available, for many applications, they are probably accurate enough to provide useful comparisons of census tracts to their municipality, host state, and the United States as a whole. In other words, they are probably accurate enough to support a typology of hazardous places and to identify some for more detailed field research. For example, the CHRR database, described earlier, ranks each county in a state from highest to lowest and compares that county with the entirety of the host state and the United States. It is an excellent place to start a research project, but it is insufficient to confidently model hazards and risks at fine geographic scales—or to confidently rank the reductions in risks that might be achieved by different investments or budget allocations in those areas.

### 3.6 Challenges in spatial statistical analysis

Assuming the data passes the accuracy test, the user confronts the following six other issues summarized in Table 2. These issues may mislead users when ignored and enhance their credibility when appropriately treated.

**TABLE 2:** Key concepts and methods in spatial data analysis.

Concepts and methods	Main ideas and refinements	Assumptions, caveats, and limitations
Spatial autocorrelation	The extent of spatial autocorrelation is important to know as: Data points close to each other in space tend to be more similar than those further apart It is fundamental to clustering and hotspot analysis	Ignoring spatial autocorrelation can lead to erroneous conclusions about data independence, affecting statistical tests’ validity
Geostatistical analysis	Methods like kriging or spatial interpolation allow estimation of values at unsampled locations based on spatial continuity measured through semivariograms by plotting the information at the known locations and fitting a model through them	Assumes stationarity and isotropy in data, which may not hold, especially across large, diverse areas. (See earlier discussion of accuracy.) All of these data represent snapshots in place and time, and analyses typically assume stationary data‐generating processes
Spatial regression	Adjusts for spatial dependence in regression models, incorporating spatial lag or error terms to account for the influence of neighboring regions	Requires careful model specification and understanding of spatial relationships to avoid omitted variable bias
Modifiable areal unit problem (MAUP)	The phenomenon where statistical results can change based on spatial aggregation level, for example, state, metropolitan region, county, municipal, census tract	Researchers need to be cautious about scale decisions; results may not be applicable at different spatial levels
Spatial heterogeneity	Recognition that relationships between variables may vary across space due to different contextual factors or local conditions, such as terrain and political boundaries	Important to consider local variation in model building; macroscale models may mask significant local differences
Network analysis	Useful for understanding connectivity and the flow of resources or information through geographical networks, that is, what is measured in areal units does not measure flows into and out of areas	Assumes that network connections are correctly represented and that important connections are not omitted from the analysis

## 4 HOW ORGANIZATIONS USE THEIR DATABASES

The United States has 3034 county governments, another 36,000 city, town, village, and township municipal governments, and 84,000 census tracts of 2000–8000 people. The 6 databases cover all of these areas, as well as almost 50,000 school districts, fire, police, utility, and other special districts. They yield well over 100 variables, and some provide US and state data for comparisons. Included are built‐in selection, analysis, and display tools. For example, suppose we wanted to know more about Flint, Michigan, and its problem with contaminated drinking water. Historically, Flint was a major automobile manufacturing center nicknamed the “Vehicle City” after General Motors was founded there in 1908. Flint's population rose from 39,000 in 1910 to 193,000 in 1970, but GM cut the workforce from 80,000 in 1978 to 8000 by 2010. Long before Flint was identified as suffering from lead‐contaminated drinking water, it had to cope with economic, environmental, and social issues and an enormous population loss. The city suffered economic crises that led to the loss of a good deal of its budget. In response to budget stresses, city officials made an ill‐fated decision to change its water source, leading to a serious decline in water quality. That major environmental challenge had repercussions not only for Flint but also for the State of Michigan and federal agencies like the EPA. The following sections discuss how we can learn about Flint and Genesee County (host county for Flint) from these six databases.

### 4.1 University of Wisconsin's public health model

The University of Wisconsin‘s CHRR database supports the university's role as an advocate for public health and reducing health‐related disparities. Users can directly access the CHRR model of health behavior at the county scale. Genesee County's population increased from 65,000 to 444,000 between 1910 and 1970 but then declined to 401,000 in 2020. Flint is the county seat of Genesee County, and its proportion of the county's population declined from 60% in 1910 to 20% in 2020.

Table 3 presents the essence of the CHRR public health model, with 6 indicators of health outcomes and 30 measures of other health factors. The weights attached to each indicator are consistent with factors influencing population health in the public health literature. Premature death, for example, powerfully reflects a set of factors that cause many people to die before age 75 years. The health factor component's highest scores are 10 for smoking and 10 for unemployment. Interactions (e.g., between smoking and obesity or between these risk factors and lack of exercise) are ignored, possibly making the implicit assumption of additive weights for different health‐related factors unrealistic. At the bottom of this list is access to exercise opportunities and the presence of dentists, each given a weight of 1. These may have low average weights when additivity is assumed yet have high importance for subsets of the population if interactions are considered. The weights represent a model of how population health influences are distributed across places, not a predictively accurate model for how changing risk factors would change health outcomes.

**TABLE 3:** Weights used in calculating health outcomes and health factors.

Health outcomes (100)	Weight, %
Length of life Premature death	50 50
Quality of life Poor or fair health Poor physical health days Poor mental health days Low birthweight	50 20 10 10 10
Health factors (100)	Weight, %
Health behaviors Adult smoking Adult obesity Food environment index Physical inactivity Access to exercise opportunities Excessive drinking Alcohol‐impaired driving deaths Sexually transmitted infections Teen births	30 10 5 2 2 1 2.5 2.5 2.5 2.5
Clinical care Uninsured Primary care physicians Dentists Mental health providers Preventable hospital stays Mammography screening Flu vaccinations	20 5 3 1 1 5 2.5 2.5
Social and economic factors High school completion Some college Unemployment Children in poverty Income inequality Children in single‐parent households Social associations Injury deaths	40 5 5 10 7.5 2.5 2.5 2.5 5
Physical environment Air pollution—particulate matter Drinking water violations Severe housing problems Driving alone to work Long commute—driving alone	10 2.5 2.5 2 2 1

*Source*: County Health Rankings & Roadmaps (University of Wisconsin, n.d.).

The raw data are measured in multiple ways. They are converted to state‐normalized *z*‐scores, where the county value minus the average county value of the host state is divided by the standard deviation of counties in the host state. Each county gets a separate health outcome and health factors score. Genesee County has the second lowest value in Michigan (ranked 82 of 83). Only Wayne County (host county for Detroit) ranks lower.

### 4.2 CDC's PLACES model

The CDC PLACES model allows users to compare Flint (rather than Genesee County) to other cities in Michigan with populations over 100,000 residents in 2010. It provides common public health metrics, such as (1) age‐adjusted asthma rates among adults, (2) age‐adjusted diabetes rates among adults, and (3) lack of insurance for those 18–64 years of age. The rates are presented with 95% confidence limits. Flint's asthma rate is higher than that of Grand Rapids, Warren, Sterling Heights, Lansing, and Ann Arbor and slightly lower than Detroit's (the 95% confidence limits do not overlap). The same pattern is true for Flint's diabetes and lack of insurance. Flint also has notably higher metric values than the State of Michigan and the United States as a whole. In other words, the PLACES database allows the user to quickly compare a specific area of interest to other places across a set of health indicators.

Similar analyses can be done in the PLACES database at the census tract scale. Results for census tract data can also be accessed through a model developed by geographer Jenks (1967) and implemented by the CDC in the 500 Cities project (CDC, 2016). The Jenks model clusters data into groups, minimizing each group's average deviation from the group while maximizing each group's deviation from the means of the other groups. That is, the method reduces the variance within groups and maximizes the variance between groups. Many readers will recognize that this is the core tool used in Fischer's linear discriminant analysis.

Jenks's method divides all US census tracts into nine classes with the range of variable values for each class indicated. The method produces an atlas of 28 maps, including 13 on health outcomes, 10 on the use of preventive services, and 5 on unhealthy behaviors for each city. Not every city has all nine groups because some cities manifest more intra‐city variation than others. For Flint, the southwest portion of the city has healthier indicators than the remainder of the city, including for asthma, diabetes, and lack of health insurance, as well as for self‐assessed mental and physical health distress. Yet all census tracts in Flint are challenged by poor health outcomes. Thus, PLACES can help users observe geographical variations across the city and census tract scales by comparing them to their surrounding areas, their host state, and the United States.

### 4.3 EPA's EJScreen and supplemental indices

The EJScreen database is a product of the EJ movement, which tends to interpret variability in outcomes in terms of injustice (e.g., poorer people may be more likely to live in poorer and dirtier areas and to have worse health than wealthier people). The US Congress heavily scrutinizes the EPA, and the agency has had to be clear about the limitations of its EJScreen tool. Its accuracy‐related limitations are mentioned in EPA's documentation (n.d.‐d), and the agency also mentions the lack of drinking water quality and indoor air quality data in its reports. EPA also carefully explains how it does and does not use the database. It informs outreach activities, enhances studies in specific places, and prepares information to support its compliance, enforcement, permitting, and voluntary programs. It also provides the data for retrospective agency reports. It is not used to (1) identify an area as an EJ community, (2) measure the cumulative impacts of multiple environmental factors, or (3) quantify specific risk values. These statements are reflected in the EJScreen tool (U.S. EPA, n.d.‐a).

The EJScreen tool does not create a single cumulative environmental score that includes all of its environmental indicators. There is no overall environmental score equivalent to the CHRR health ranking and outcome scores for each county. Instead, EPA provides supplemental indices for 13 of its environmental indicators, including PM2.5, ozone, toxic releases to the air, traffic proximity, lead paint (housing age as a surrogate), Superfund, and hazardous waste proximity. Environmental indicators are not weighted, but there is weighting by demographic markers of presumed social justice. The 13 supplemental indices are calculated by multiplying the environmental indicator percentile for the area (e.g., block group) by demographic indices (low‐income percent, unemployed percent, limited English‐speaking percent, less than high school percent, and low life expectancy rate). For example, if users were interested in underground storage tanks, the environmental number for the area would be multiplied by the demographic indicators. This presumably reflects a policy or belief that EJ requires considering demographics rather than counting all people equally, regardless of their characteristics.

In the case of Flint, we examined the 13 supplemental indices using EJScreen. The area in southwest Flint had relatively low values for underground storage tanks, proximity to hazardous waste sites, and high lead paint (representing older housing stock). Higher values marked the city's northernmost area. In other words, the EJScreen indices identified environmental hazards that are more marked in areas with populations assumed to have higher vulnerability. Although there is no cumulative indicator of EJ‐weighted environmental risks in EJScreen, users can create cumulative indicators by downloading the data and calculating a single value using their definitions and formulas. By recognizing the limitations of the data and not assigning weights to the metrics, the EPA provides data and allows communities to decide how to respond accurately and what to do about weighting the information. The agency is careful not to declare areas with high values as EJ communities.

### 4.4 FEMA's National Risk Index (NRI)

Substantial increases in economic damage in the United States from hurricanes, tornadoes, and other natural hazard events in recent decades led FEMA to develop a practical and easy‐to‐use risk tool for natural hazard mitigation planning. The NRI (FEMA, n.d.) is a cumulative number calculated for counties and census tracts that consider 18 natural hazards, the expected annual losses associated with them, the vulnerability of the people and structures in the area, and likely local resilience. FEMA makes the NRI data user‐friendly by converting the granular data into proportions compared to the host state and the United States as a whole.

The online database is easy to query, and the intended primary users are the groups that prepare local hazard migration plans. This includes local officials and employees, planners, real estate professionals and insurance agents, research groups including academics, and property owners. The NRI database has historical data and assigns economic value to damage, as well as the likelihood of an occurrence. The data are used to update emergency plans, tweak zoning and building codes, address risk‐related gaps in requests for financial support for capital structures, identify needs for full‐fledged risk assessments, and construct communication plans for high‐risk events.

Looking at the data for Genesee County in Table 4, the 89.3 means that it has a higher NRI risk value than more than 89% of counties in the United States and 96% of counties in Michigan. Social vulnerability for Genesee County is higher than 68% of US counties and 92% higher than Michigan ones. Finally, community resilience is 72% in the United States and 47% in Michigan. In other words, Genesee is more resilient than most Michigan counties but about average for US counties. The FEMA database indicates that Genesee County is highly vulnerable to extreme winter weather typically associated with proximity to the Great Lakes.

**TABLE 4:** Hazard risk data for Genesee county, Michigan.

	Genesee county is % higher than		
Indicator	United States counties	Michigan counties	Hazard threats: Values in the highest 20% compared to the United States
Risk index	89.3	96.4	Cold wave, ice storm, lightning, strong wind, and tornado
Expected annual loss	89.3	95.2	
Social vulnerability	67.7	91.6	
Community resilience	71.9	47.0	

*Note*: Higher numbers imply higher risk for the risk index, expected annual loss, and social vulnerability. Higher numbers for community resilience indicate more resilience.

*Source*: National Risk Index (FEMA, n.d.).

The NRI county and city hazard risk data are necessary inputs to hazard mitigation plans, which states, counties, and larger municipalities produce to be eligible for disaster relief support from the federal government. The FEMA database and analyst tools do not predict the risk‐reduction benefits that hazard mitigation plans would create. Rather, the quantitative NRI data are added to assessments from committees that prioritize risks after broad consultation with the local governments and the public. Note that the data in Table 4 are entirely consistent with the county's list of concerns for infrastructure failures, loss of communications, electricity, potable water, sewage, and roads. Otherwise stated, FEMA's risk model produces easily understood information for natural hazards, which can advise local analysts and lead to the need for specific risk assessments.

### 4.5 ATSDR's Environmental Justice Index (EJI)

ATSDR's (2022) EJI is a compilation of 36 variables used to measure community risk, as defined by specific value weights applied to data elements (Table 5). This linear scoring approach ignores interactions among factors in causing risks. The 36 measures include indicators from EPA's databases, the Census Bureau, CDC's PLACES, the US Mine and Safety and Health Administration, and TomTom Multinet (a company that prepared data for transportation and park access‐related issues). The data are divided into environmental burden and social and health vulnerability modules (Table 5). Each of the three modules receives a score, which is then converted to a cumulative score ranging from 0 to 3. The final score is a percentile ranking from 0 to 1. Thirty of the 36 indicators in EJI or equivalents are used in the EPA, University of Wisconsin, CDC, and FEMA databases. In other words, the ATSDR EJI index uses much of the same data but with a different goal in mind. The goal is to provide a starting place to identify distributive and EJ and their impacts on human health and well‐being.

**TABLE 5:** Indicators used by the environmental justice index model.

Indicator module and group	Indicator
Social vulnerability	
Racial/Ethnic minority status	Minority status
Socioeconomic status	Poverty No high school diploma Unemployment Housing tenure Housing‐burdened lower‐income households Lack of health insurance Lack of broadband access
Household characteristics	Age 65 and older Age 17 and younger Civilian with a disability Speaks English “less than well”
Housing type	Group quarters Mobile homes
Environmental burden	
Air pollution	Ozone PM 2.5 Diesel particulate matter Air toxics cancer risk
Potentially hazardous $ toxic sites	National priority list sites Toxic release inventory sites Treatment, storage, and disposal sites Risk management plan sites Coal mines Lead mines
Built environment	Lack of recreational parks Houses built re‐1980 Lack of walkability
Transportation infrastructure	High‐volume roads Railways Airports
Water pollution	Impaired surface water
Health vulnerability	
Pre‐existing chronic disease burden	Asthma Cancer High blood pressure Diabetes Poor mental health

*Source*: Table adapted from ATSDR (2022).

ATSDR notes that the EJI is not a definitive indicator of EJ communities. However, the results lead to such an application. An indicator is flagged if it is the 75th percentile or higher. It does not require much effort to add up the high‐ranking values as a cumulative score.

The average EJI ranking for Flint as a whole was 88, that is, the city as a whole has a higher cumulative risk (as defined by the selected scoring system) than 88% of communities in the United States. Tract 36, located on the southwest edge of the city, has lower values for poverty and many other indicators than the city as a whole, but it is not better off than most other census tracts in the United States. Where 50 corresponds to the central tendency values for US census tracts, the tract of 6557 people has an overall EJ rank of 95, which includes ranks of 75 or more for older housing; transportation related to high‐volume roads and a short distance from the county airport; its impaired water system; lack of internet access; civilians with a disability; and children aged 17 or younger. The tract's biggest concentration of high cumulative risk values is for socioeconomic status, including scores ranging from 76 to 98 for poverty, less than HS graduation, unemployment, and housing burden for low‐income people, as well as for health outcomes and behaviors (asthma, high blood pressure, diabetes, and poor mental health). Overall, half of the indicators are flagged and in the high group, which is the case for nearly all the tracts in Flint. EJI delivers a stark message about Flint and its census tracts by using these red flags. Although the US public focuses on the drinking water issue, the number of values even higher than the value of 88 for the water system stands out. The relative cumulative risk for those living in Flint is clear compared to other places in Michigan, but this tool is not meant to calculate an actual community or personal risk.

### 4.6 Council on environmental quality's CEJST

CEJST is probably the most important of the six databases because it provides the primary input used by federal agencies to distribute federal grant dollars under the Justice40 program. The database draws factors from a variety of categories that influence human health risk outcomes and burdens. Additionally, CEQ has shown a willingness to listen to and adjust to a chorus of suggestions from a variety of sources. A remaining issue, however, is the use of arbitrary thresholds to categorize a census tract as eligible or not eligible as disadvantaged. These differ for each category of disadvantage. Consider the threshold for being eligible as a disadvantaged community due to climate change. The CEJST website states that:

Communities are identified as disadvantaged if they are in census tracts that are at or above the 90^th^ percentile for expected agriculture loss OR expected building loss rate OR expected population loss rate OR projected flood risk OR projected wildfire risk (CEQ, n.d.).

Such a definition has the limitation that a community above the 90th percentile for expected agriculture loss AND expected building loss rate AND expected population loss rate AND projected flood risk AND projected wildfire risk receives the same classification—“disadvantaged”—as does a community with none of these difficulties in a census tract that has just one of them. Resource allocations based on such categorizations may not distinguish between communities that would benefit greatly from risk‐reducing resources and communities that would benefit somewhat less (see also Benson et al., 2023).

Poverty and other demographic indicators are gatekeepers for entry into eligibility for the Justice40 program. Dean and Esling (2023) estimate that 37% of US census tracts and 32% of the US population are considered disadvantaged. Densely settled areas in cities typically have higher rates of poverty, high proportions of African American residents, and 10 or more exceedances compared to rural areas. The break occurs when six or more threshold criteria are exceeded. Below six, less of the population is poor and non‐Hispanic White. CEQ has partly responded to the criticism that it was more difficult for rural areas to be eligible by allowing eligibility based on exceeding one environmental criterion (Dean & Esling, 2023). Although the current version of CJEST allows space for less disadvantaged rural places to be included, how the criteria are used to influence funding in the future will be a political decision. That is, so many US census tracts are classified as “disadvantaged” that we should expect political influence as well as risk estimates to be used to determine how funding is distributed (Greenberg & Schneider, 2019).

Every census tract in Flint is included as disadvantaged by CJEST, and Detroit is part of the same disadvantaged inner‐city urban process. Yet, the most striking concentration of disadvantaged areas in Michigan is in the northern part of the state (north of Midland), in rural areas with losses of jobs and attractions that would otherwise retain working‐age people (Jesse, 2019). The CJEST database sharply demonstrates that Michigan's oldest automobile manufacturing centers and its northern rural areas have become places of considerable poverty and abandonment.

## 5 USER‐INITIATED APPLICATIONS

The six US taxpayer‐funded (some foundations have funded several of these databases) covered by this tutorial can help users examine risk‐related questions that are not directly part of their organizations’ missions. We provide one example initiated by the first author and then a table that suggests others.

### 5.1 Location of medical marijuana sites

Are legally sanctioned medical marijuana sites disproportionately found in neighborhoods with existing social and EJ hazards or simply in places with excellent market access? An interesting geographically centered challenge evolving in the United States during this decade has been locating legal cannabis distribution centers. The US federal government considers cannabis an illegal substance. Yet, many states have legalized it and are creating state‐specific management policies. An increasing share of US residents favor legalization for medical purposes and recreation. Gallup surveys (Jones, 2022) show that in 1969, only 12% of the US population favored legalizing marijuana. In 1977, the proportion rose to 28% and then to 50% in 2011. By 2021, the proportion was 68%. Jones (2022) concludes that only self‐declared conservatives, mostly aged 65 and older, oppose legalizing marijuana. Dioun (2018) argues that cannabis supporters evoked the argument that cannabis was needed to help seriously ill people to open the door to the recreational marijuana industry. Trends are for rapidly increasing demand. Flowhub (2022) data estimate that sales increased 134% between 2019 and 2021.

Watching the evolving geography of cannabis distribution centers is fascinating in so far as it raises multiple ethical questions, including risk‐related ones, such as where sites should be located. This is an important question because some argue that marijuana is a benefit to many people and can bring jobs to some neighborhoods. Others argue that selling cannabis brings unsavory people to neighborhoods and devalues property. California was the first US state to site cannabis distribution facilities. Studies of siting locations in California, New York, and Washington State all show mixed results. Although there was a relationship with markets, some but not all showed a relationship with environmental and social justice issues (Amiri et al., 2019; Cunningham et al., 2022; Gruenewald, 2011; Jones et al., 2018; Morrison et al., 2014; Tabb et al., 2018; Will et al., 2021; Wall et al., 2011).

Two major concerns exist when considering the locations of legal cannabis centers: (1) economic centrality and (2) environmental and social justice. The former suggests that legal medical marijuana sites should be located in densely populated neighborhoods along major highways readily accessible to customers. The latter claims that licensed cannabis distribution centers are disproportionately located in neighborhoods with locally unwanted land uses (LULUs), environmental and public health challenges, and high concentrations of people of color and low socioeconomic status. By November 2022, the State of New Jersey licensed 23 cannabis distribution facilities. We used EJScreen to test the 23 sites for the market and justice issues. Two variables in EJScreen measured centrality: traffic proximity and population density. Eight variables measured presumed aspects of social and EJ: percent people of color; low income; less than a high school education; limited English‐speaking family; the unemployment rate; low life expectancy; Superfund proximity; and underground storage tanks. If centrality was the driver for site location, then the sites would be disproportionately located in areas with a relatively high population density and near roads with high traffic density. If social and EJ were the locational drivers, many of the eight categories should be disproportionately found near the cannabis dispensaries.

The flexibility of EJScreen's database was apparent in this test. Census tracts are not acceptable data accumulation areas because of their wide range in shape and population sizes (Li et al., 2018). Some census tracts are ellipses, others resemble triangles and rectangles, and still others wind along physical features. Furthermore, census tracts vary in size and population. In some areas, they may reflect as few as 1000 persons. In some large cities, they may contain more than 20,000. As there is no consistency in the size or shape of census tracts, after some preliminary testing, we set EJScreen to employ a standard 1‐mi circle (3.14 mi^2^) around the cannabis dispensaries as the representative choice. This allowed us to apply the same geographic definition across all 23 dispensary locations.

Comparing each of New Jersey's 23 cannabis site areas to other locations was another advantage of EJScreen. In addition to a 1‐mi radius around each site, we also examined four of New Jersey's most distressed cities that did not have a cannabis dispensary location (Camden, Newark, Passaic City, and Trenton). The city group was created by aggregating the results for all eight metrics for the four cities and weighting them by each city's population size. A third comparison was with the dispensaries’ host county. For example, Essex County was the county for the City of Newark. The expectation was that the 1‐mi radius around the cannabis distribution sites would be most similar to the 3‐mi radius areas around the site and notably different from their host counties and the four distressed cities.

To test centrality, we examined EJScreen maps for the locations of the 23 legal cannabis distribution centers in New Jersey. With one exception, each was within 1 mi of an interstate road or state highway, often near the intersection of two roads. The maps showed that most were in existing retail locations on major roads. Bars and restaurants were visible nearby, but so were pharmacies, grocery stores, and churches. The maps provided preliminary evidence supporting the economic centrality theme. Analysis of the centrality variables showed the 1‐mi radius areas around the 23 sites all had a population density of less than half of the distressed cities, and they were almost 60% higher than their host counties. Traffic density was 30% less than in the distressed cities and only 11% higher than in the 3‐mi radius areas surrounding the cannabis sites. All of these differences were statistically different at the conventional alpha = 0.05 significance level, with matched‐pair *t*‐tests at *p* < 0.05. In other words, the cannabis distribution sites are near and accessible to potential markets, thereby supporting the centrality theme.

For the social and EJ indicators, all eight comparisons had lower median values for the 1‐mi areas than in the four distressed cities (median 38), all significantly different at *p* < 0.05. In sharp contrast, the cannabis distribution areas were similar to their surrounding 3‐mi areas and their host counties. Across the eight justice indicators, the median ratio for the host counties was 1.01, and for the 3‐mi areas, it was 0.94. In other words, a relationship supporting the locations of cannabis distribution centers with LULUs and demographic inequalities was not found.

In short, we found the first 23 cannabis distribution sites in New Jersey located for their centrality to markets, and they did not seem to influence environmental and social justice. Of course, there is a chance that as the market expands for the recreational use of marijuana, location rules will change the siting processes.

This case study on marijuana exemplifies the potential utility of using publicly available datasets to proactively monitor neighborhood change. The data were available at the census tract scale, and they were reset to provide uniform‐size circular study areas. This process removed spatial bias, allowing evaluators to see what is happening in places of the same size and shape. Used proactively, these databases and their tools can be used to anticipate the impact of different site choices on demographic and LULU‐related outcomes. They can provide early warnings that can direct developers away from some locations. They can also be used to help planners better understand the equity implications of location choices and monitor the outcomes of various locational options. Overall, the medical marijuana case study illustrates the flexibility of the EJScreen database to address a specific policy challenge that was not anticipated when the databases were designed.

### 5.2 Other options and challenges they raise

The number of opportunities for users to employ these databases is exciting. We briefly touch on several in Table 6.

**TABLE 6:** Summary of other applications with analysis and discussion.

Scenarios	Analysis	Discussion
Assessing environmental justice (Using EJScreen)	Combine demographic and environmental hazard data to identify areas where minority and low‐income communities may face disproportionate environmental risks	Environmental data may not reflect actual exposures, so conclusions about environmental injustice should be tentative and verified with ground‐level data. This limitation is crucial as errors in environmental data can lead to misallocated resources and policies that fail to address real risks. See Mohai et al. (2009) on the flaws of using proxies in environmental justice assessments. See also Willemain et al. (2003) and Pannell and Gibson (2015)
Planning for public health (using CHRR and PLACES)	Analyze health outcomes with socioeconomic and environmental data at city and county levels to identify priority areas for health interventions	Aggregating health data at different scales can mask localized health issues; small area analysis is recommended where data permits. Granularity in data reporting is needed so as not to obscure acute local problems and underfunding. See Schuurman et al. (2009), Deeth and Deardon (2016), and Greenberg and Schneider (2023)
Natural disaster risk assessment (using FEMA's NRI)	Utilize the NRI to map out economic and social vulnerabilities to natural disasters at the census tract and county levels to aid in disaster preparedness and mitigation planning	Estimates based on historical data might not accurately predict future risks due to changing climate patterns. Reliance on historical patterns without accommodating for climate change trends can skew risk assessments. See Cutter et al. (2010). See also Peterson and Vose (1997) and Maraun et al. (2010)
Evaluating community resilience (using CEJST)	Apply the CEJST to assess community vulnerability to climate‐related and environmental hazards for guiding resource allocation under the Justice40 initiative	Classifying communities as “disadvantaged” may oversimplify the dynamics of environmental and social resilience and lead to ineffective policy formulations. See Maantay (2002) and Jones (2024)

Abbreviations: CEJST, Climate and Economic Justice Screening Tool; CHRR, County Health Rankings and Roadmaps; NRI, National Risk Index.

### 5.3 Scanning for new opportunities and developments

Risk assessment is a dynamic field. It seems highly likely that artificial intelligence (AI) and machine learning (ML) will be increasingly deployed to help automate and improve some parts of research, such as identifying relevant literature that may have been overlooked and checking statistical modeling results, methods, and assumptions. These new tools should allow for deep learning, low‐dimensional embeddings of high‐dimensional data, and other sophisticated methods to find additional statistical connections among observed variables. For example, Schmidt (2020) and Adefemi et al. (2023) point to rapid expansion in the use of AI to monitor air and water quality, disaster response infrastructure, and diseases. This capability offers the opportunity to better understand geographical patterns but also raises the threat of causal relationships being claimed based on correlations and potentially misleading interpretations (Shimonovich et al., 2021.; U.S. EPA, n.d.‐e). Such errors can create deeply misguided public policy. Ongoing vigilance is imperative to prevent algorithmic bias that steers policy in the wrong direction. AI is improving our capability, but we need more experience before turning monitoring over to it.

A call for new data quality standards is a second issue we should expect. Section 3.1 opens with a discussion of existing US efforts. These efforts will continue, but the rapid evolution of data gathering, storing, and releasing information is going to lead to data obsolescence, questions about the precision of data, the adequacy of data sources, and data quality control and quality assurance. Users will need to scan for changes in data collection practices and understand their limitations. For example, we can now generate synthetic data, that is, data created by models. We noted earlier that EJ Screen's numbers for particulate air quality, ozone, diesel particulates, and others are produced by using monitoring fixed station data and kriging to estimate values for places without monitors. Although these data are valuable, they should be used with caution. Reality may hold some surprises that real data could reveal, but synthetic data cannot because the models used to generate the data do not capture those unexpected features.

Hypermaps feature the ability to integrate text, images, audio, video, and animation. Users can navigate multimedia datasets by theme but also spatially. MacEachren and Kraak's (1997) edited book offered insights into how data visualization could evolve; as it has turned out, this evolution has been even more rapid than anticipated, and we can now look at risk‐related issues at some sites through multiple lenses. However, the evolution of data visualization for one or more risks at many locations is problematic. Census data go back well over a half‐century, which allows for tracing social and economic changes. The same is not true for environmental monitoring data, which has changed methods and locations. EPA has some sites that have been used for decades, yet many other sites have been relocated, which undermines historical studies at fine geographical scales.

This article has focused on publicly available data. Private organizations collect proprietary data, some of which would be extremely helpful for measuring risk. Accessing these data, however, might be problematic and costly. We view data sharing as an important challenge that has been evolving as owners of public companies call for more information (Eagan et al., 2001; U.S. GAO, 2020).

Lastly, we note the opportunities presented by large datasets available beyond US borders. Data Europa (European Union, 2024) has over 1.4 million datasets from 36 countries in six languages. From among well over 100 publications, we recommend Schmidt et al. (2020) as an entry point for understanding the massive amount of information collected and how it can be accessed. We also recommend a conference paper by EU staff that describes the development of their database systems for the environment (Hřebíček et al., 2015).

Understanding how global data are collected and how they are revised is key to doing good analyses. For instance, the United States provides considerable data for an average census tract (approximately 4000 people). In contrast, Managi and Li (2023) explain how Japan's 500‐m grid system provides population data for the period 2001–2020. In other words, although there is much to be learned by periodically examining what different nations are doing, doing cross‐national analyses is challenging.

## 6 CONCLUSIONS AND RECOMMENDATIONS

Unless the US government and the public decide to limit funding for these large databases or limit public access to them, their number will grow, and the sets of variables they include will markedly expand. This will allow for more investigations of the geographies of hazards and presumed indicators of EJ. It is plausible that cuts could occur to the budgets that support these databases, which would be a setback and frustrate potential users. In the long run, it is hard to believe that such constraints will stop progress on publicly available databases.

Researchers face an ongoing challenge of wisely using the wave of new databases illustrated in this article, and teachers need to reinforce their limitations with students. Table 7 highlights some of the main concerns and possible ways to overcome them.

**TABLE 7:** Concerns, misinterpretations, and possible paths forward for spatial data utilization.

Concerns	Examples of misinterpretations	Possible paths forward
Accuracy and reliability of spatial data	Misinterpretations of flood risk maps in urban planning have led to inadequate infrastructure and disaster preparedness, notably in New Orleans pre‐Hurricane Katrina	Advances in real‐time data collection and machine learning are improving data accuracy and reliability. Continuous updating of spatial databases can help mitigate misinterpretations by providing more current and refined data assessments
Spatial scale and resolution	In health studies, using broad spatial scales to infer air quality impacts has resulted in policies that fail to address localized pollution sources (e.g., asthma rates in inner cities vs. suburban areas)	Increasing availability of high‐resolution spatial data and tools like GIS allows precise mapping at fine scales, potentially leading to more targeted and effective policy interventions
Ethical use of spatial data	The use of racially biased policing data in predictive policing algorithms has led to disproportionate targeting of minority communities, reinforcing systemic inequalities	There is growing awareness and debate about ethical data use. Initiatives like open data policies and ethical guidelines are being developed to guide the responsible use of spatial data, ensuring fairness and equity in data‐driven decisions
Integration of temporal dynamics	Historical crime data used to allocate police resources often do not take into account the dynamic nature of urban crime, leading to over‐policing in areas that may no longer be high risk	The integration of temporal dynamics into spatial data systems is becoming more common, with time‐series analysis and dynamic modeling techniques enabling more adaptive and responsive policy planning
Impact of data granularity on decision‐making	Overgeneralization of economic data in rural development projects has often led to inappropriate resource allocation, such as the misdirected funds in Appalachian economic development programs	The push for micro‐scale data in spatial analysis is helping refine development strategies that are better tailored to the specific needs of smaller communities or regions. Enhanced data granularity is leading to more nuanced understanding and decision‐making

In summary, these six databases present opportunities to better understand the geography of risk and hazards in the United States. Their role is as a screening tool, and as such, they must be used with caution. We end with the following seven suggestions for those working with large spatial databases:

1. Identify specific research questions that are supported by a body of theory and ideas that are plausible and consistent with empirical tests. What, if anything, regarding causality is implied or assumed by the questions?
2. Examine the database background documents to determine the developers’ objectives and assumptions and how these are expressed in the variables, as well as their temporal and geographical scales. Are there important gaps in the databases? For example, did they fail to collect data in three states for 2 years? Can the gaps be filled? Is there a workaround for the gaps? Overall, do the databases allow a fair examination of the questions?
3. Examine databases for opportunities to compare places. Do the databases include places that can be used for comparisons (e.g., nation, state, local government, census tracts‐blocks)? Do the databases allow the user to create polygons that are a better fit for answering the research questions than are standard politically defined areas?
4. Examine accuracy‐related limitations of the available data. Read data quality reports to learn how point data was assigned to areas. Look for missing data and explanations for missing data. Should some of the data be converted from interval and ratio scales to ranks and categories to accommodate uncertainty and inaccuracy concerns?
5. What statistical and AI/ML tools/models are appropriate to answer the questions? Can the tests be performed with more than one method to prevent the result from being dependent on a single method? Can the assumptions behind the tests be verified for the data?
6. Do the existing databases have built‐in tools that will allow the questions to be answered inside the database? Is it possible to test the results with a sample before using the entire database? Do the results make sense for the places with extreme values?
7. If other statistical tools are needed, can the data be exported to those tools? How much time will it take, and what is the estimated cost?

## Data Availability

There is only one data analysis section, and it used publicly available data.
